# Current and Future Aspects of Multimodal Imaging, Diagnostic, and Treatment Strategies in Bicuspid Aortic Valve and Associated Aortopathies

**DOI:** 10.3390/jcm9030662

**Published:** 2020-03-01

**Authors:** Shazia Afzal, Kerstin Piayda, Oliver Maier, Shouheng Goh, Katharina Hellhammer, Mareike Cramer, Florian Bönner, Amin Polzin, Niels Nijhof, Malte Kelm, Tobias Zeus, Verena Veulemans

**Affiliations:** 1 Division of Cardiology, Pulmonology and Vascular Medicine, Heinrich Heine University, Medical Faculty, Moorenstr. 5, 40225 Düsseldorf, Germany; Shazia.Afzal@med.uni-duesseldorf.de (S.A.); Kerstin.Piayda@med.uni-duesseldorf.de (K.P.); Oliver.Maier@med.uni-duesseldorf.de (O.M.); Shouheng.Goh@med.uni-duesseldorf.de (S.G.); Katharina.Hellhammer@med.uni-duesseldorf.de (K.H.); Mareike.Cramer@med.uni-duesseldorf.de (M.C.); Florian.Boenner@med.uni-duesseldorf.de (F.B.); amin.polzin@med.uni-duesseldorf.de (A.P.); malte.kelm@med.uni-duesseldorf.de (M.K.); zeus@med.uni-duesseldorf.de (T.Z.); 2 Philips Healthcare, 5684 Best, The Netherlands; Niels.Nijhof@philips.com; 3 CARID (Cardiovascular Research Institute Düsseldorf), Moorenstr. 5, 40225 Düsseldorf, Germany

**Keywords:** aortopathy, congenital heart disease, bicuspid aortic valve

## Abstract

Bicuspid aortic valve (BAV) is the most frequent congenital cardiac abnormality leading to premature aortic valve apparatus dysfunction and is often associated with aortopathy. Therefore, current guidelines recommend a surgical aortic valve replacement (SAVR), even if many patients are deemed inoperable owing to their comorbidities and require alternatives such as transcatheter aortic valve replacement (TAVR). However, BAV variations remain challenging for procedural success. Therefore, the latest development in different imaging modalities (echocardiography, multislice-computertomographie, cardiovascular magnetic resonance) allows in-depth analysis for preprocedural risk stratification, follow up, and patient selection. Furthermore, we shed light on the latest developments in pre- and periprocedural fusion imaging as well as on current and future treatment options.

## 1. Introduction

The bicuspid aortic valve (BAV) is the most common congenital anomaly in adults. The frequency of this congenital aortic valve defect is given as approximately 1–2% in the total population, whereby regional differences need to be taken into consideration [1]. When evaluating the overall risk of all possible complications, the importance of BAV disease is higher than that of all other congenital heart abnormalities combined [2]. In contrast to a tricuspid aortic valve (TAV), BAV is a heterogeneous disease presenting with different phenotypes and complications [3]. To date, the ontogenetic development of the BAV anatomy as well as the progressive calcifications of the aortic valve is not fully understood. The following different hypotheses have been discussed. The malformation of the aortic cusps takes place during valvulogenesis. Hereby, either the cusps fail to separate owing to abnormal blood flow during valvulogenesis or a secondary fusion happens, leading to different phenotypes. It has been shown that aortic stenosis develops more often if the aortic cusps are asymmetrical or in the anteroposterior position. Furthermore, BAV may also be induced as a result of genetic defects encoding endothelial nitric oxide synthase, as shown in mice models. Besides, oxidative-mechanical stress leads to an activation of osteogenesis, and thus enhances the process of valve calcification. Another hypothesis leading to degeneration of the aortic media is that the level of cellular matrix metalloproteinases (enzymes diminishing matrix) is elevated in the aorta of patients with BAV [4]. Owing to the great variability of the BAV anatomy, imaging methods are particularly important in diagnostics and therapy. There are different classifications for the varying bicuspid valve morphologies. On the basis of the presence and characteristics of the raphe, commissural position, description of the cusp and its size, and the aortic sinus characteristics, the most commonly applied classification is by Sievers and Schmidtke. Hereby, the valve morphology is determined by the number of cusps and the presence of raphes, as well as the position and symmetry of the cusps [5]. Another classification was introduced by Jilaihawi et al. [6]. The authors describe three types—a tricommissural type, a bicommissural raphe type, and a bicommissural non-raphe type—providing a deeper understanding of the interaction of any valve prosthesis with the native aortic valve at the basal leaflet plane and at the commissural level. Tricommissural type was the name given for a functional BAV without aortopathy owing to rheumatic, fibrotic, or calcifications leading to a commissural fusion.

BAV leads to an accelerated damage of the valve apparatus, mostly beginning at a younger age and resulting in a more rapid severity progression than in TAV. Furthermore, BAV is commonly associated with aortic pathologies (e.g., aneurysms, dissections, and coarctations) and, therefore, a larger diameter of the ascending aorta as compared with the normal population with TAV. This thoracic aortic dilation is detected in approximately 40% of patients [1]. The dilatation of the aorta can affect different sections of the ascending aorta and occur very differently in its shape or configuration. Owing to the enlarged diameter of the ascending aorta, there is evidence of an increased risk of aortic dissection and aortic aneurysms.

Although the current guidelines recommend a surgical aortic valve replacement (SAVR) for BAV, there are patients who are deemed inoperable owing to their comorbidities and require alternatives such as transcatheter aortic valve replacement (TAVR). Due to the challenging large anatomical variety in BAV, asymmetric calcifications, coronary anomalies, and associated aortopathies, multimodal imaging is pivotal for adequate patient selection and risk stratification concerning different treatment approaches. Therefore, we reviewed the role of different imaging tools for the diagnosis and treatment in this specific heterogeneous disease as well as current and future treatment options.

## 2. Diagnostic Approaches

### 2.1. Aortic Valve Morphology and Function

**Figure 1:**
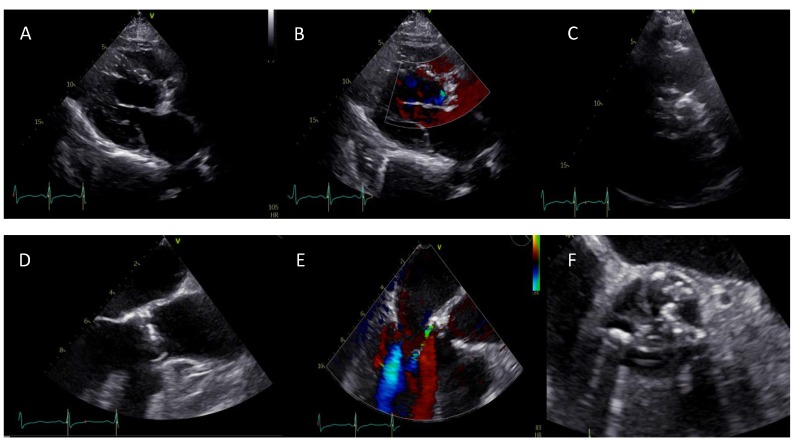
(**A**–**C**) Initial evaluation by transthoracic echocardiogram of a 49-year-old male patient. Parasternal long axis view reveals a calcified aortic valve with restricted valve opening as well as a regurgitation jet. Parasternal short axis en-face view shows a severely calcified aortic valve with a bicuspid anatomy. However, the precise type could not be identified because of the image quality. (**D**–**F**) Transesophageal echocardiography (TEE) revealed a bicuspid valve type 1 R/N morphology (Sievers) and a mild aortic regurgitation with an eccentric jet.

Different imaging modalities are of importance in the diagnosis of BAV and possible complications. Echocardiography is the recommended imaging method of choice to visualize the valve anatomy and function. Aortic stenosis (AS) is observed more frequently in BAV than aortic regurgitation. An initial assessment by transthoracic echocardiography (TTE) based on peak jet velocity, mean transvalvular gradient, and effective orifice area measured according to the continuity equation is recommended. A subgroup may reveal severe AS with a tight valve area, but low transvalvular gradient. This might be associated with impaired left ventricular function or reduced stroke volume in a highly hypertrophied left ventricle of restrictive cardiomyopathy. Moreover, BAV is often combined with relevant aortic regurgitation. Its assessment relies on color, continuous, and pulsed wave Doppler accompanied with the left ventricular function. In some cases, TTE cannot sufficiently determine the direction of the aortic valve insufficiency jet in the longitudinal axis view (central or eccentric) and its origin in the short axis view (central or commissural) [7] (Figure 1). The bicuspid aortic valve can lead to an eccentric flow jet that can be better detected with transesophageal echocardiography (TEE), especially if patients have left ventricular hypertrophy or left ventricular dysfunction. Therefore, TEE may be preferred in patients with aortic insufficiency. Parameters obtained through three-dimensional (3D) echocardiography, tissue Doppler, and strain rate imaging may be useful, especially in patients with reduced left ventricular ejection fraction (LVEF), to determine the optimal time for intervention [8].

**Figure 2:**
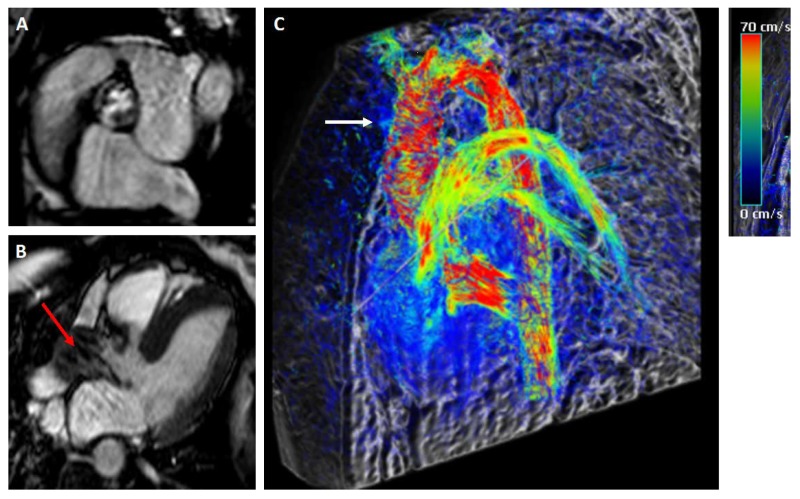
Twenty-four-year-old female patient after surgical resection of a coarctation of the aorta and ligation of Ductus Botalli. A concomitant bicuspid aortic valve disease was shown as well. A recent cardiovascular magnetic resonance (CMR) exam revealed hemodynamics of a calcified aortic valve with moderate aortic valve stenosis (AVA 1.1 cm^2^, transvalvular velocity 3.9 m/s). (**A**) A typical SSFP cine CMR still frame giving a perspective from the left anterior side of the left ventricle to the aorta in systole. (**B**) Three-chamber (LVOT) view SSFP cine still image demonstrating the restricted leaflets and the narrow jet of high velocity (red arrow). (**C**) Time-resolved 3D phase contrast—4D flow—demonstrated a vortex flow (white arrow) in the ascending aorta during systole.

According to class I recommendations, cardiovascular magnetic resonance (CMR) should be used to determine the backflow volume if echocardiographic measurements are ambiguous [9]. The different imaging modalities assess the anatomical progression of the bicuspid aortic valve and usually only record the anatomical alterations of the vessels and heart valves and not the rheological load on the aortic wall. Using the results from 2D phase contrast (PC) CMR studies, some research groups evaluated the hemodynamically forces on the aortic wall using 4D flow [10]. In contrast to 2D PC-CMR, the 3D PC-CMR with three-directional velocity encoding (4D flow) comprises information on the in vivo 3D blood flow dynamics with full volumetric data set of the thoracic aorta and time in the cardiac cycle. Furthermore, 4D flow enables a retrospective blood flow analysis at any region of interest within the 3D data set. For the quantification, standard parameters such as peak and mean velocities, total flow, net flow, retrograde flow as viscous energy loss, wall shear stress, and turbulent kinetic energy can be assessed. In contrast to 2D PC-CMR assessment with simplified Bernoulli equation, 4D flow calculations of pressure gradients in a vessel segment (measured in three directions) are based on the Navier–Stokes equation, presuming incompressionable laminar Newtonian fluid flow. Owing to the laminar flow assumption, the assessment of AS can be challenging because of its turbulent flow. First studies have shown that, compared with 2D Doppler echocardiography, 4D flow assessment is similar with its three-directional velocity field measurements [11]. Further same day echo-CMR studies are required investigating the advancements of these technical refinements, especially in the assessment of AS [12] (Figure 2). However, severe bulky calcifications may challenge the diagnosis of BAV and BAV-derived complications. Therefore, multi-slice computed tomography (MSCT) is the gold-standard imaging technique to assess the distribution and extent of aortic valve calcifications.

### 2.2. Aortic Valve Sizing

Precise imaging examinations are of importance concerning the planning before interventional or surgical intervention in order to prevent severe complications such as prosthesis dislocation, annulus rupture, and severe paravalvular leakage. According to the current guidelines, MSCT is the preferred imaging tool for assessing the anatomy and dimensions of the aortic root, size, and shape of the aortic valve annulus; its distance from the coronary ostia; the distribution of calcifications; and the number of aortic valve leaflets. 3D-TEE can be used to determine aortic valve annulus dimensions, but is more operator and image quality dependent than MSCT [13]. Compared with TAV, BAV often demonstrates a larger annulus size and Sinus of Valsalva with increased dimensions of the ascending aorta. BAV annulus is less elliptical and reveals asymmetrical calcifications. In contrast to TAV anatomy, in BAV, it can be challenging to define the annulus plane. There is no consensus on the appropriate methodology for BAV sizing. Until now, there are different techniques that have been suggested such as annulus-based sizing, and supra-annular sizing measuring the intercommissural distance [14,15,16].

### 2.3. BAV-Associated Aortopathy

An 80-fold higher risk of aneurysms compared with the normal population could be demonstrated where the ascending aorta is affected in 60–70% of the cases [17]. When measuring the aortic root, the TTE measurements are comparably lower than those measured with an ECG-controlled MSCT. However, all cut-off criteria for interventions are currently based on echocardiography data. If root dilation by TTE is suspected, further assessment with MSCT or CMR should be performed to clarify the findings. Both, MSCT or CMR scan, are recommended to assess the presence and extent of aortopathy [1]. CMR can be used for postprocedural assessment, but the indication for surgery should preferably be based on MSCT measurements. Choosing between MSCT and CMR depends on their availability and the age of the patient. CMR is recommended for patients under the age of fifty, avoiding MSCT-associated radiation exposure. MSCT or CMR should be applied in patients with severe aortic dilation of at least 45 mm owing to possible treatment indications. It must be mentioned that bulky calcifications of the ascending aorta can only be sufficiently determined by MSCT, offering a contraindication for surgical treatment. Table 1 gives an overview of the advantages and disadvantages of the several imaging modalities.

## 3. Treatment Options

The most important clinical decision is the optimal time for SAVR or TAVR. The maximum aortic diameter, the presence of aortic risk factors such as connective tissue disorders, and the presence of surgical risk factors (e.g., advanced age, reduced left ventricular function) and accompanying indications such as aortic valve stenosis or insufficiency must be taken into account.

### 3.1. Surgery

Surgical treatment consists of aortic valve replacement using a biological or mechanical prosthetic heart valve. The selection of the heart valve prosthesis (i.e., biological or mechanical) fundamentally depends on the age of the patient and some other factors, and should be determined in a detailed patient informed consent before the planned operation [18]. Current guidelines [18,19] recommend surgery with an aortic diameter of 50 mm if at least one of the following risk factors is present: aortic coarctation, systemic hypertension, familial aortic dissection, or rapid aortic growth of more than 3–5 mm/year. In certain cases, even with a smaller aortic diameter, an indication for surgery can be given, namely in patients with a small body surface or stature, and in patients with Turner syndrome, as these patients are at increased risk of aortic complications. In BAV patients with an indication for aortic valve surgery, simultaneous aortic replacement from an aortic diameter of at least 45 mm is recommended, as a higher incidence of subsequent aortic events such as aortic dissection could be demonstrated in retrospective studies. The type of valve implanted determines the extent of aortic repair. A complete aortic root replacement is advisable in patients with a mechanical valve, while in young patients with a biological valve, the risk of a later aortic root rupture is low, so a root replacement is not necessary. There is not yet enough evidence to deal with an aortic arch aneurysm associated with the bicuspid aortic valve. If a patient with an increasing aortic aneurysm and a normal aortic diameter is below the outflow of the truncus brachiocephalicus, an increasing aortic repair without arch intervention is recommended. If the aortic arch at the start of the brachiocephalic trunk has a diameter of more than 45 mm, the replacement of the hemiarch is reasonable. A complete arch replacement is advisable with an average aortic arch diameter of at least 45 mm [1].

### 3.2. Transcatheter Aortic Valve Replacement (TAVR)

TAVR is well known to be the treatment option of choice in inoperable and high-risk patients, and currently even shows favorable outcome in intermediate and low-risk cohorts with TAV [18,19,20]. Until now, TAVR in BAV has largely been excluded from randomized clinical trials owing to concerns about calcification-related under-expansion of the device, probably leading to a more relevant paravalvular leackage (PVL), the risk of annulus rupture in a concomitant aortopathy, and coronary occlusion, as well as concerns about long-term durability [21]. Therefore, it appears unlikely that industry-sponsored randomized trials will be established in the future. Nonetheless, the expansion of TAVR to low-risk patients will remain a challenge in BAV anatomy and associated aortopathy as well as those aged <60 years in decision making of the multidisciplinary heart-team. Currently, there are no guideline recommendations and very limited data exit regarding the impact of the aortic aneurysm in TAVR. Interestingly, case reports have demonstrated successful transapical TAVR in combination with thoracic endovascular aortic repair in a patient with 8.0 cm ascending aneurysm and 4.2 cm aortic arch aneurym as a possible approach in high-risk inoperable patients. Furthermore, it has been reported that there was no increase of the ascending aortic aneurym after TAVR in BAV anatomy [22].

**Figure 3:**
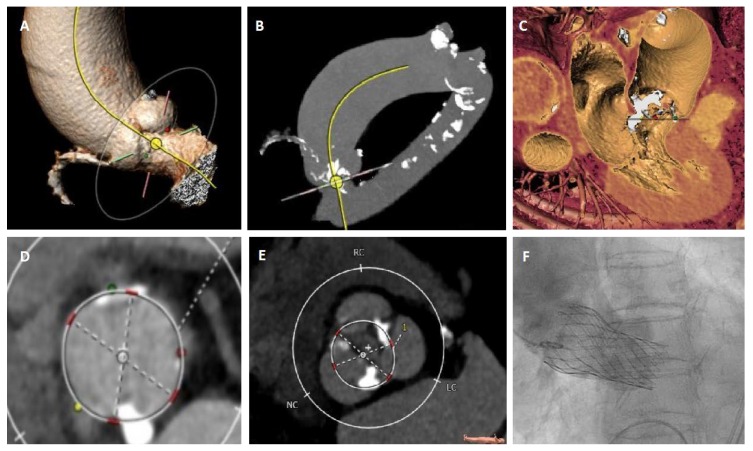
(**A**,**B**) MSCT diagnostics revealed a bicuspid aortic valve type I L/R with a concomitant aneurysm (42 × 47 mm) and (**C**) severe eccentric calcification of the left ventricular outflow tract. (**D**) Annulus sizing at the level of the native annulus (perimeter 72.5 mm; area 417 mm^2^). (**E**) Supra-annular sizing (perimeter 71.5 mm; area 396 mm^2^). (**F**) Successful implantation of an under-sized self-expandable valve (CoreValve Evolut R 26 mm) owing to the highly calcium load in the LVOT and aortic aneurysm.

Looking into the limited data, both balloon and self-expandable prostheses can be implanted successfully in selected BAV anatomies. However, a higher rate of more than mild perivalvular leak was found among patients treated with self-expandable valves [23]. A higher incidence of annular rupture was demonstrated in the group of patients treated with a balloon-expandable valve, while most of the study data concerning efficiency and outcome were realized using old-generation devices [5,24,25,26]. Oversizing of the transcatheter valves can lead to an under-expansion of the bioprosthesis, leading to a significant PVL. This can be improved with post-dilatation management, which likewise increases the risk of annular rupture and conduction disturbances. Therefore, under-sizing and a high implantation depth may be considered in specific anatomies based on calcification burden and the narrowest supra-annular sizing. Figure 3 offers an example of TAVR with a self-expandable device in a BAV with concomitant aortopathy.

However, new-generation transcatheter valves significantly improved the outcomes of TAVR in BAV in concerns of peri-procedural complications and early- to mid-term outcomes [27,28,29]. Nonetheless, long-term durability of transcatheter valves as well as concomitant aortopathy should be considered, while an accompanying relevant aortic aneurysma cannot be resolved by interventional techniques. Therefore, future studies are needed to evaluate the progression of ascending aorta aneurysm or late aortic dissection/rupture in patients with BAV undergoing TAVR [30].

## 4. Future Perspectives

### 4.1. Diagnostic Modalities

The number of TAVR procedures is expected to increase significantly in the following years. Improving efficacy will become essential for experienced operators in high volume centres, while less experienced operators will require in-depth training. Hereby, especially the BAV morphologies will remain challenging and require accurate pre- and peri-procedural work up. An accurate automated detection of aortic root dimensions might not only increase efficiency, but also reduce operator variability and the impact of the experience of the operator on sizing.

Currently, there are two approaches described based on preprocedural MSCT data. Astudillo et al. described a deep learning method detecting the aortic annulus automatically and comparing to a control group that performed sizing manually. The automatically obtained device size selection was fast, accurate, and reproducible [31]. Another group validated a preprocedural patient-specific computer simulation based on MSCT data regarding the occurrence of PVL or conduction abnormalities in a small cohort of patients and compared the data with postprocedural imaging modalities such as MSCT, echocardiography, and fluoroscopy for bicuspid aortic valve morphologies. They could demonstrate that there was an accurate correlation in perimeter and area. Using a computer simulation affected the clinical outcome such as a reduction of the occurrence of paravalvular leakages and conduction abnormalities [32].

**Figure 4:**
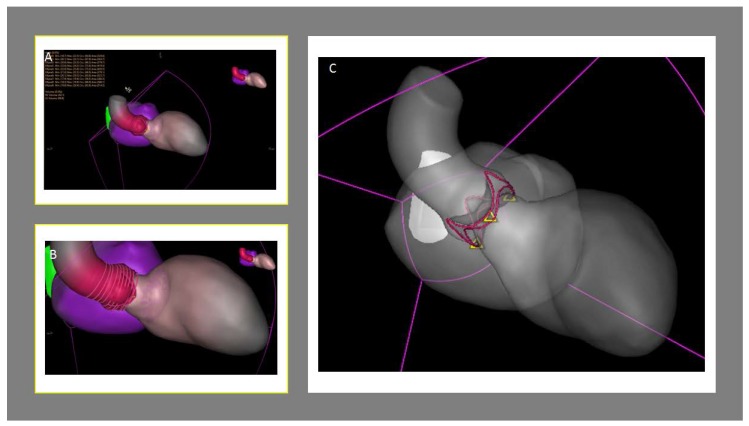
An investigational device of next-generation of real-time echocardiography-fluoroscopy fusion imaging (EchoNavigator) allows, based on a 3D echocardiographic dataset, the establishment of a 3D heart model. (**A**,**B**) The 3D heart model is automatically co-registered to the patient. Once the patient-specific 3D heart model has been generated, sizing of the aortic valve (not validated) can be exported from the system. (**C**) Moreover, the interventionalist can rotate the C-arm to a position where all three hinge points of the several leaflets are aligned in one plane. It can predict the optimal angulation for transcatheter aortic valve replacement (TAVR) prosthesis deployment and needs to be evaluated further.

Next-generation of real-time fusion of echocardiography and fluoroscopy enables a patient-specific segmented 3D heart model, which is feasible for any structural heart disease interventions. On the basis of a high-quality 3D TEE image of the aortic valve and its surrounding structured and a successful ECG-gated segmentation, a patient-specific 3D heart model can be obtained. As 3D TEE annulus measurements are comparable with MSCT in selected patients (e.g., such as chronic kidney disease or challenging morphologies that require periprocedural TEE), automated sizing may be performed intra-procedurally (Figure 4). This approach has not been validated yet and the question arises whether it will be implemented in daily routine. While low-volume centers require TEE guidance at the beginning of their learning curve, one needs to take into consideration that more and more high-volume centers have shifted from general anesthesia to conscious sedation over the past years in order to minimize the risk of delirium and cognitive disorders in elderly patients with many comorbidities [33].

In order to improve communication among the interventionalists and the understanding of 3D spatial orientation the application of holography for imaging was recently introduced. Holographic images allow a real-time visualization of a 3D-patient-specific dataset based on MSCT (RealView Imaging Inc., Yokneam, Israel) and/or CMR data (CarnaLife Holo, MedApp S.A., Krakow, Poland) floating in the air during the procedure in front of the interventionalists, enabling an intuitive and interactive display of the complex anatomy. Rymuza et al. describe its use in TAVR during a BAV anatomy type I L/R, facilitating the navigation and implantation of the valve prosthesis. Further studies are needed to evaluate its impact, for example, on the procedure-related parameters (procedure length, amount of contrast agent, radiation exposure) [34,35,36,37].

3D-printing of the heart is a relatively new tool with high potential in planning complex surgical and transcatheter-based interventions, especially in congenital heart diseases [38]. It is an enlightening diagnostic application, including educational (anatomic understanding and valve-design) as well as practical (surgical and interventional) aspects. Until now, a widespread use is restricted to high costs, printing time, and heart-circle-derived anatomic deformation processes. However, 3D-printing may change the diagnostic and interventional work-up to a heart-model-based individual and intelligent treatment in complex anatomies.

### 4.2. Treatment Options

Temporal trends and outcomes of transcatheter versus surgical aortic valve replacement in BAV revealed similar results on complications and outcomes [39]. However, several approaches over standard biological or mechanical valve replacement demonstrated new possible treatment strategies. Concerning surgical strategies, several aortic valve reconstruction techniques have been described for the repair of BAV, including flail segment resection of the prolapsing leaflet, resection of the raphe, and annuloplasty approaches. However, the durability of BAV repair is unclear [40,41]. The ROSS-procedure, utilizing a pulmonary autograft as aortic valve replacement, has been improved in the past, and is thus not a new technique at all, but considered rare in clinical practice owing to the technical complexity [42]. While most of the patients treated by the ROSS-procedure are younger [43], post-operative aortic root expansion can be prevented through stabilization with a prosthetic Dacron graft [44]. Recently published long-term results are encouraging, showing favorable hemodynamics, quality of life, and freedom of re-surgery after nine years of follow-up [45,46].

Tricuspidization, as a technique of turning a bicuspid into a tricuspid valve, is a novel technique, agitating enhanced attention in surgical repair for adults [47,48] and TAVR according to BASILICA-techniques [49] with favorable hemodynamic results. Further developments of these techniques may address challenges like non-circular device expansion, worse device durability, and PVL in BAV-related aortic stenosis in the future.

**Figure 5:**
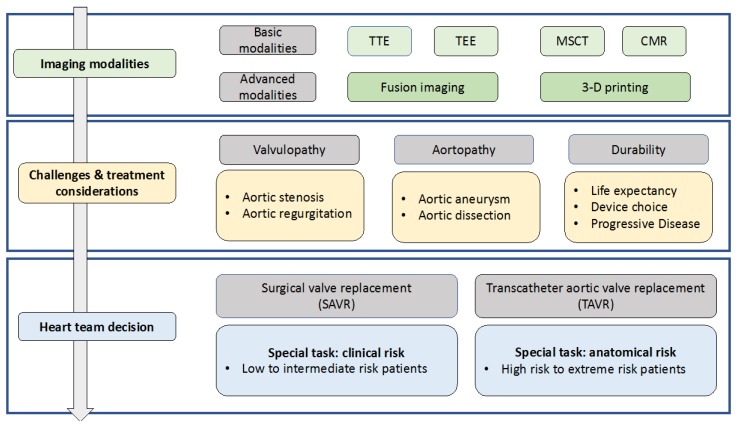
Imaging modalities, specific considerations and treatment strategies in bicuspid aortic valve (BAV). TTE, transthoracic echocardiography.

It must be pronounced that TAVR for BAV-related pure aortic regurgitation is not mentioned in the current guidelines and was listed as a contraindication before. However, there are several high-risk patients who cannot undergo surgical treatment. Off-label TAVR in pure aortic regurgitation has to face important challenges like necessary oversizing for anchoring without calcium; the associated risk of dislocation, annular rupture, and PVL; as well as frequently observed too large anatomies for current available TAVR devices. However, TAVR is likely to be established as an applicable alternative treatment with new dedicated devices in time [50]. Figure 5 gives an overview on the diagnostics, challenges, and important considerations in the context of treatment allocation.

## 5. Conclusions

Multimodal imaging in bicuspid aortic valve is essential for diagnosis and treatment of the progression of valve dysfunction and associated aortopathy. Besides the optimal time of surgical intervention, TAVR as an alternative treatment will gain more importance as it expands to low-risk patients and will include bicuspid aortic valve pathologies and new treatment strategies. Advanced pre-procedural fusion imaging tools may strengthen diagnostic accuracy and support best treatment strategies in complex anatomies.

## Figures and Tables

**Table 1:** Advantages and disadvantages of different imaging modalities for bicuspid aortic valve (BAV) and BAV-related aortopathy diagnosis. TTE, transthoracic echocardiography; TEE, transesophageal echocardiography; CMR, cardiovascular magnetic resonance.

Modality	Indication	Advantages	Disadvantages
TTE	- aortic valve function - aortic valve morphology - aortic dimensions	- cost-effective and widely available - initial assessment - no exposure to radiation - 3D echo assessment	- depends on imaging specialists experience - limited by imaging window
TEE	- aortic valve function - aortic valve morphology - aortic dimensions, aortopathy - assessment of aortic annulus dimensions	- no exposure to radiation - 3D echo assessment	- conscious sedation - invasive - limited visualization of coronary arteries and aortic arch - experienced imaging specialist
MSCT	- aortic valve morphology - aortic dimensions, aortopathy - assessment of aortic annulus dimensions - alcification of aortic valve	- short scanning time - excellent evaluation of calcifications and quantification - high spatial resolution—not limited by imaging window	- exposure to radiation—not able to provide information on aortic valve or left ventricular dysfunction - limited availability - contrast usage - need of breath-hold
CMR	- aortic valve morphology - aortic dimensions, aortopathy - aortic valve function - evaluation of left and right ventricular function	- no exposure to radiation - not limited by imaging window - tissue characterisation - hemodynamic assessment - evaluation of myocardial tissue with late gadolinium enhancement - multi-parametric assessment	- limited availability - end-stage renal failure - claustrophobia requiring sedation - implanted metallic devices—arrhythmia - long scanning time - need of breath-hold - no efficient evaluation of calcifications
